# Editorial: Targeted Genome Editing in Crops

**DOI:** 10.3389/fgeed.2021.757916

**Published:** 2021-09-09

**Authors:** Huanbin Zhou, Yong Zhang, Lanqin Xia, Seiichi Toki

**Affiliations:** ^1^ Institute of Plant Protection, Chinese Academy of Agricultural Sciences, Beijing, China; ^2^ Department of Biotechnology, School of Life Sciences and Technology, University of Electronic Science and Technology of China, Chengdu, China; ^3^ Institute of Crop Sciences, Chinese Academy of Agricultural Sciences, Beijing, China; ^4^ Department of Plant Life Science, Ryukoku University, Otsu, Japan; ^5^ Institute of Agrobiological Sciences, National Agriculture and Food Research Organization (NARO), Tsukuba, Japan; ^6^ Kihara Institute for Biological Research, Yokohama City University, Yokohama, Japan

**Keywords:** genome editing, gene targeting, CRISPR, cas protein, gene function study, crop improvement

In the past 4 decades, tremendous progress has been made in our understanding of plant molecular biology, especially the molecular basis of a wide range of agronomic traits, such as seed development, nutrient acquisition, stress tolerance, disease resistance, etc. However, compared to the active researches in model plants, our current knowledge about crops lags far behind due to the limited availability of mutants for gene function study. In addition, translational research, which applies the fundamental knowledge into improved agronomic traits in crops, is also poor due to technical problems. Fortunately, the newly-developed, cutting-edged genome editing technologies, which enable precise sequence modifications in the crop genomes, provide effective tools to address these challenges. Nowadays, various loss-of-function mutants and novel gain-of-function alleles of genes of interest can be easily generated *via* genome editing. These new gene editing technologies bridge the gaps between gene function study and crop improvement.

So far, engineered meganucleases, zinc finger nucleases (ZFNs), transcription activator-like effector nucleases (TALENs), and clustered regularly interspersed short palindromic repeats (CRISPR)-CRISPR-associated protein (Cas) have been successfully adapted for targeted genome editing in crops. Of these, the CRISPR/Cas systems, characterized by the RNA-guided DNA nucleases, have been widely employed in a large number of crop species for multiple applications since it is simple-to-design, easy-to-use, and highly-efficient. Generally speaking, the CRISPR/Cas nucleases generate a double-strand DNA break at the target site in the crop genome, resulting in indel mutations *via* the error-prone non-homologous end joining pathway or DNA fragment replacement in the presence of donor template through the homology-directed repair pathway (Sukegawa et al., 2021). In addition, different kind of effectors can be engineered with CRISPR/Cas nickase system for precise nucleotide editing. For example, cytidine deaminases or adenine deaminases could be guided by CRISPR/Cas to the target sequences, inducing C to T and A to G conversion, respectively (Ren et al., 2018; Yan et al., 2018), and CRISPR/Cas9-fused M-MLV reverse transcriptase could introduce all possible nucleotide substitutions and combinations through reverse transcription (Li et al., 2020; Tang et al., 2020).

In this special topic of frontiers in genome editing, we have compiled the recent advances in the development and applications of targeted genome editing technology in crops. Ten original research papers on the special topic of genome editing, including DNA fragment replacement, gene knockout and base editing, in a number of crops are included in this issue. Seiichi Toki’s lab present two powerful gene targeting (GT) methods utilizing a SSA (Single-Strand-Annealing)-mediated marker excision system and a CRISPR/Cas9-mediated strategy with all-in-one vector. In their first paper, Ohtsuki et al. used SSA-mediated marker excision system to introduce three and seven multiple discontinuous bases into the microRNA miR172 target site of *OsCly1* gene in rice to achieve the cleistogamous flowering phenotype. In their second paper, Nishizawa-Yokoi et al. developed a CRISPR/Cas9-mediated GT strategy utilizing a single, all-in-one vector containing a CRISPR/Cas9 expression construct, a selectable marker and a donor template. In their study, Nishizawa-Yokoi and colleagues utilized this novel system for homology-directed repair (HDR) in rice and tobacco, successfully modifying specific target genes such as *OsALS*, *NtALS*, *NtEPSPS*, etc.

Oz and colleagues from Fredy Altpeter’s lab present novel advances in sugarcane gene editing. In their study, Oz and colleagues present a detailed description on the generation of herbicide-tolerant sugarcane plants by co-editing multiple alleles of *ALS via* HDR-mediated repair of double-strand DNA break induced by CRISPR/Cas9.

PAM compatibility is a limiting factor in the CRISPR/Cas9 system. To address this issue, Zhang and colleagues from Jinxiao Yang’s lab provide an evidence that tRNA-esgRNA broadens the PAM compatibility of xCas9 nuclease, enabling it to function at NG, GAA, GAT, and GAG PAM sites in rice. Moreover, Zhang and colleagues further enrich the CRISPR toolbox by developing an xCas9-based cytosine base editor (CBE) capable of editing NG and GA PAM sites.

Bruyn and colleagues’ paper brings into light what is required for efficient gene modification of witloof. In their study, Bruyn and colleagues report a highly-efficient CRISPR/Cas9-mediated genome editing workflow in traditional Belgian crop witloof based on PEG-mediated protoplast transfection, whole plant regeneration and HiPlex amplicon sequencing. By using this platform, Bruyn and colleagues successfully edit *CiGAS*, *CiGAO*, and *CiCOS*, which are of significant importance to control the biosynthesis of sesquiterpene lactones and hence the bitterness of witloof.

*Nicotiana benthamiana* is one of the most utilized model plant species in plant molecular biology. Thus, the paper presented by Hsu and colleagues from Choun-Sea Lin’s lab is a noticeable contribution in this research area. In their paper, Hsu and colleagues report a simple, highly robust genome-editing in *N. benthamiana* protoplasts by using SaCas9, SpCas9, FnCas12a, and nCas9-based CBE, and successfully regenerated stable lines from protoplast. Moreover, Hu and colleagues report that CBEs can enable precise C-to-T substitutions at endogenous loci in rapeseed.

To facilitate the generation of transgene-free edited lines, He and colleagues from Yunde Zhao’s lab report a simple method to detect transgenic events using a visual marker. In their study, He and colleagues develop an anthocyanin-marker assisted CRISPR system that enabled the identification of transgene-free and target gene-edited plants in T1 generation based on anthocyanin accumulation.

Advances in the use of CRISPR/Cas in *N. tabacum*, rice and tomato are also presented in this special issue. Donovan et al. and Lin et al. describe the successful application of the CRISPR/SpCas9 system in editing *rbcS* homologs in *N. tabacum* and *PP2A-1* in rice. Finally, Vu et al. presents a comprehensive review on the latest developments in precision genome editing in tomatoes and prospects its applications in breeding.

CRISPR is a technology which is constantly evolving. As presented in the articles on this special issue, CRISPR/Cas is developing into a “game-changing” technology in plant science with diverse applications ranging from basic studies to the more applied translational research. We hope that the topics covered in this issue will boost the benefit of genome editing technology, not only for gene discovery but also for crop improvement.
